# Syntheses of ^15^N-labeled pre-queuosine nucleobase derivatives

**DOI:** 10.3762/bjoc.10.199

**Published:** 2014-08-18

**Authors:** Jasmin Levic, Ronald Micura

**Affiliations:** 1 Institute of Organic Chemistry, University of Innsbruck and Center for Molecular Biosciences Innsbruck, Innrain 80–82, 6020 Innsbruck, Austria

**Keywords:** heterocycles, ligands, nucleic acids, nucleobases, nucleosides, pyrrolopyrimidinones

## Abstract

Pre-queuosine or queuine (preQ_1_) is a guanine derivative that is involved in the biosynthetic pathway of the hypermodified tRNA nucleoside queuosine (Que). The core structure of preQ_1_ is represented by 7-(aminomethyl)-7-deazaguanine (preQ_1_ base). Here, we report the synthesis of three preQ_1_ base derivatives with complementary ^15^N-labeling patterns, utilizing [^15^N]-KCN, [^15^N]-phthalimide, and [^15^N_3_]-guanidine as cost-affordable ^15^N sources. Such derivatives are required to explore the binding process of the preQ_1_ base to RNA targets using advanced NMR spectroscopic methods. PreQ_1_ base specifically binds to bacterial mRNA domains and thereby regulates genes that are required for queuosine biosynthesis.

## Introduction

**Scheme 1:**
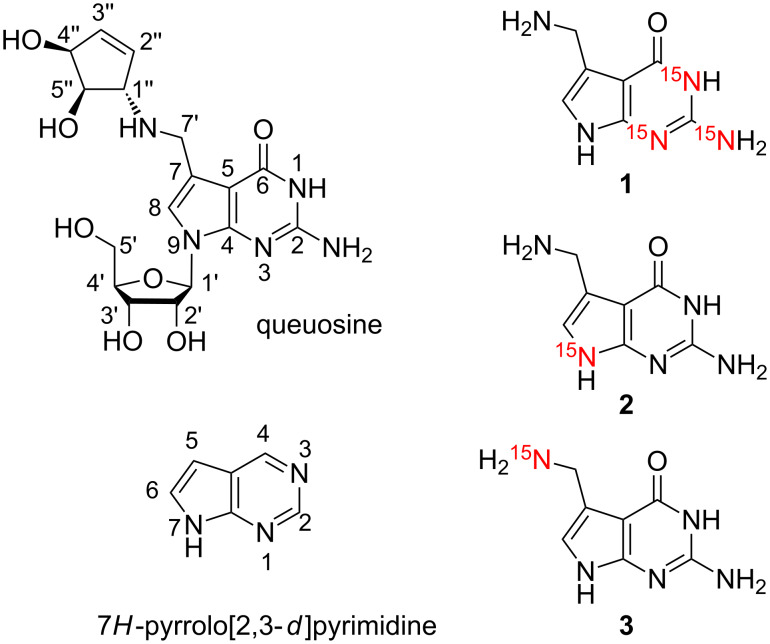
The hypermodified nucleoside queuosine (Q) and the synthetic targets of preQ_1_ bases **1** to **3** with complementary ^15^N labeling patterns for potential NMR spectroscopic applications. Purine and systematic numbering as indicated.

The small pyrrolo[2,3-*d*]pyrimidine 7-(aminomethyl)-7-deazaguanine is a natural product, also termed prequeuosine base (preQ_1_ base) [1–2]. This guanine derivative is involved in the complex biosynthetic pathway of the hypermodified tRNA nucleoside queuosine [3]. Recently, preQ_1_ base has attracted considerable attention because this nucleobase specifically binds to bacterial mRNA domains and regulates genes that are required for queuosine biosynthesis, by a so-called riboswitch mechanism [4–8]. To explore the binding process of preQ_1_ base to the RNA and to shed light on the dynamics underpinning this process advanced NMR spectroscopic methods exist for which ^15^N-labeled preQ_1_ base derivatives would be highly beneficial. Here, we report efficient routes for the synthesis of three derivatives with complementary ^15^N-labeling patterns (Scheme 1).

The synthesis of preQ_1_ base has been described first in 1979 by Goto and coworkers from 2-methylthio-6-methoxy-7-methyl-7-deazapurine in 13 steps [9]. Another early, but more efficient procedure was reported by Nishimura in 1988 based on the Mannich reaction using dibenzylamine–formaldehyde and 2-acylaminopyrrolo[2,3-*d*]pyrimidin-4(3*H*)-one, which resulted in the selective introduction of the dibenzylaminomethyl group [10]. The following amine exchange reaction of the dibenzylamine function in the Mannich base with ammonia resulted in the preQ_1_ base. More recently, Carell and coworkers developed a straightforward pathway based on the key reaction of in situ α-brominated 3-phthalimidopropanal with 2,6-diaminopyrimidin-4-one [11], inspired by Grubb’s synthesis of the Q base (queuine) [12]. Alternatively, Klebe and coworkers employed a Michael addition of the same pyrimidinone to the nitroolefin 2-[(2*E*)-3-nitroprop-2-en-1-yl]-1*H*-isoindole-1,3(2*H*)-dione [13], however, this route seemed inconvenient for our purposes because access to the nitroolefin requires several additional steps.

## Results and Discussion

Our aim was to develop a robust synthetic pathway to the preQ_1_ derivatives with the three complementary ^15^N labeling patterns depicted in Scheme 1. For this undertaking we considered Carell’s synthesis [11] of preQ_1_ base as a solid foundation that we intended to adapt and modify accordingly, under the premises of efficacy and cost-minimization for ^15^N incorporation.

**Scheme 2:**
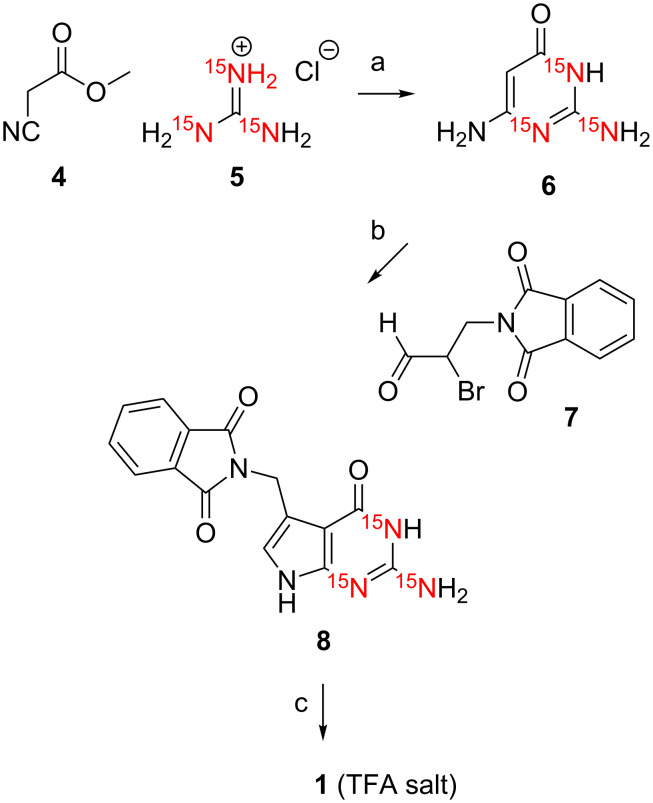
Synthesis of [^15^N1,^15^N3,H_2_^15^N(C2)]-preQ_1_ base (**1**). a) CH_3_ONa (10 equiv) CH_3_OH, reflux, 10 h, RP C18 chromatography, 35%. b) NaOAc·3H_2_O (2 equiv), acetonitrile/water, 40 °C, 4 h, 63%. c) H_2_N-NH_2·_H_2_O (10 equiv), ethanol, reflux, 14 h, RP C18 chromatography, 75%. Compound **1** was isolated as salt of trifluoroacetic acid (TFA).

For [^15^N1,^15^N3,H_2_^15^N(C2)]-7-(aminomethyl)-7-deazaguanine (**1**), we started with the reaction of methyl cyanoacetate (**4**) and [^15^N_3_]-guanidine hydrochloride (**5**) under basic conditions to give the corresponding [^15^N1,^15^N3,H_2_^15^N(C2)]-2,6-diaminopyrimidin-4-one (**6**) in high purity after work-up and reversed-phase column chromatography (C18) (Scheme 2) [14]. Then, the α-bromo aldehyde **7** was obtained in two steps from commercially available 3-phthalimidopropan-1-ol that was oxidized using Dess–Martin periodinane. Subsequent in situ bromination of the 3-phthalimidopropan-1-al with CH_3_SiBr, described previously by others [11–12], did not work reliable in our hands.

Therefore, in anlogy to Grubb [15] and a more detailed protocol by Yamaguchi [16], we applied 5,5-dibromobarbituric acid [17] to obtain the α-bromo aldehyde **7** which was well stable during purification by column chromatography on SiO_2_ and isolated in good yields. The pyrrolo[2,3-*d*]pyrimidine ring system of preQ_1_ base was built in good yields via the cyclocondensation reaction between [^15^N1,^15^N3,H_2_^15^N(C2)]-2,6-diaminopyrimidin-4-one (**6**) and the 2-bromo-3-phthalimidopropan-1-al (**7**). Finally, deprotection was performed with hydrazine hydrate. The previously published route [11] recommended *N*-Boc functionalization of the preQ_1_ base in the crude reaction mixture to enable flash chromatography on SiO_2_ followed by cleavage of the auxiliary function, however, although robust in handling, the yields were rather modest. We therefore decided to directly purify the crude product by reversed-phase column chromatography (HPLC) and obtained compound **1** in excellent yield and purity. We mention that compound **1** was isolated as salt of trifluoroacetic acid (TFA) using 1% TFA in the eluent and it was assumed to exist in 1:1 stoichiometry (preQ_1_:TFA) based on ^1^H NMR spectra and consideration of p*K*_a_ values. However, it is noteworthy that a crystal structure of the preQ_1_·TFA salt that was crystallized from saturated aqueous solution showed the co-existence of mono- (N(C'7)) and dications (N(C'7), N3) in the crystal [11].

**Scheme 3:**
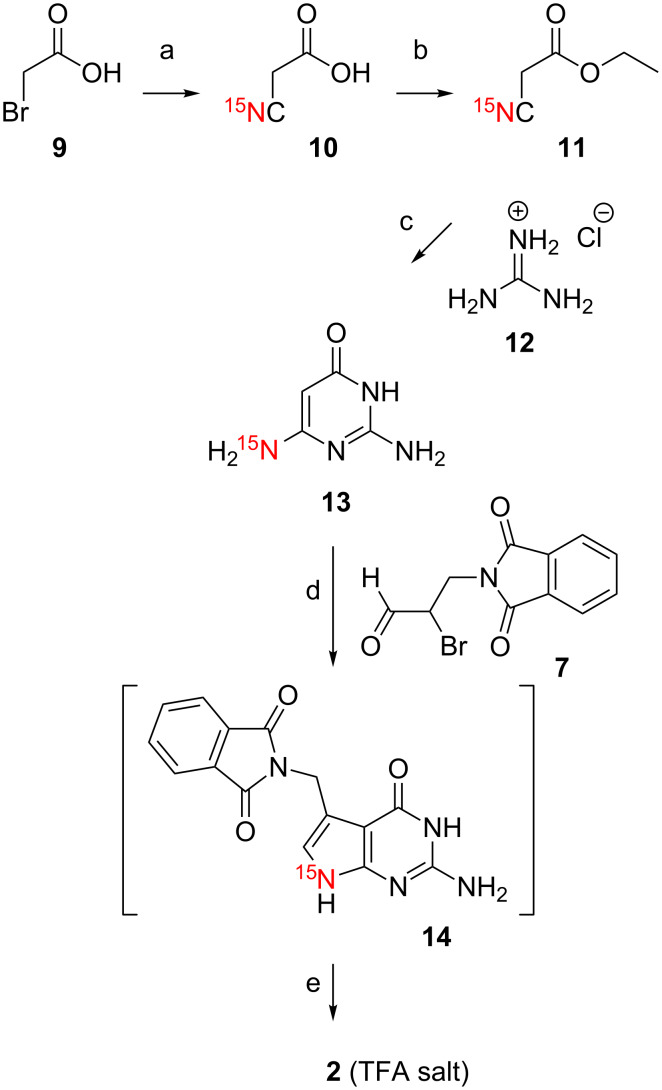
Synthesis of [^15^N9]-preQ_1_ base (**2**). a) [^15^N]-KCN (1 equiv), Na_2_CO_3_, H_2_O, pH 9, 80 °C, 3 h, then room temperature, 20 h, 90%. b) Ethanol (5 equiv), H_2_SO_4_ cat., reflux, 18 h, 92%. c) CH_3_ONa (10 equiv) CH_3_OH, reflux, 10 h, RP C18 chromatography, 40%. d) NaOAc·3H_2_O (2 equiv), acetonitrile/water, 40 °C, 4 h, 52%. e) H_2_N-NH_2·_H_2_O (10 equiv), ethanol, reflux, 14 h, RP C18 chromatography, 92%. Compound **2** was isolated as salt of trifluoroacetic acid (TFA).

The synthetic track for the [^15^N_3_]-preQ_1_ base (**1**) was designed with the concept in mind to access the complementary ^15^N patterns of [^15^N9]-preQ_1_ base (**2**) and [H_2_^15^N(C7')]-preQ_1_ base (**3**) by employing the same key steps. In this sense, the key intermediate [H_2_^15^N(C6)]-2,6-diaminopyrimidin-4-one (**13**) for target **2** was accessible by first synthesizing ethyl [^15^N]-2-cyanoacetate (**11**) from 2-bromoacetic acid (**9**) and potassium cyanide [^15^N]-KCN, followed by esterification (Scheme 3).

All further steps were conducted in direct analogy as described for target **1**, namely reaction with guanidine hydrochloride to furnish compound **13**, followed by cyclocondensation with 2-bromo-3-phthalimidopropan-1-al (**7**) to give the protected [^15^N9]-preQ_1_ base **14** for subsequent deprotection yielding the desired [^15^N9]-preQ_1_ base (**2**) (Scheme 3).

**Scheme 4:**
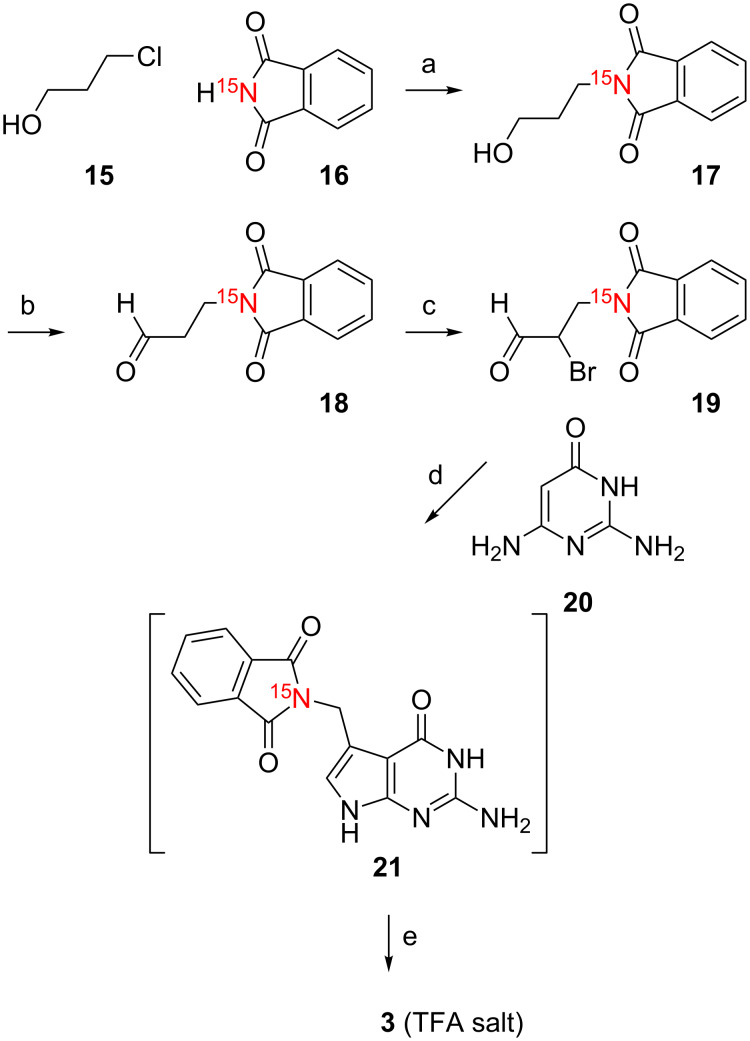
Synthesis of [H_2_^15^N(C7')] preQ_1_ base (**3**). a) K_2_CO_3_ (1.5 equiv), DMF, 70 °C, 14 h, 47%. b) Dess–Martin periodinane (1.5 equiv), CH_2_Cl_2_, 3 h, room temperature. c) 5,5-dibromobarbituric acid (0.6 equiv), acetonitrile, reflux, 2 h, 45%. d) NaOAc·3H_2_O (2 equiv), acetonitrile/water, 40 °C, 4 h, 58%. e) H_2_N-NH_2·_H_2_O (10 equiv), ethanol, reflux, 14 h, RP C18 chromatography, 82%. Compound **3** was isolated as salt of trifluoroacetic acid (TFA).

Also for the third target, [H_2_^15^N(C7')]-preQ_1_ base (**3**), our strategy turn out to be highly convenient. First, we prepared the ^15^N-labeled aldehyde **18** as the key intermediate (Scheme 4). This was achieved by reaction of 3-chloropropanol (**15**) with [^15^N]-phthalimide **16** to give [^15^N]-3-phthalimidopropan-1-ol (**17**). All further steps were in direct analogy as described for targets **1** and **2**, namely reaction with 5,5-dibromobarbituric acid [17] to obtain [^15^N]-2-bromo-3-phthalimidopropan-1-al (**19**), followed by cyclocondensation with commercially available 2,6-diaminopyrimidin-4-one (**20**) to give the protected [^15^N(C7')]-preQ_1_ base **21** for subsequent deprotection yielding the desired [^15^N(C7')]-preQ_1_ base (**3**) (Scheme 4).

**Figure 1:**
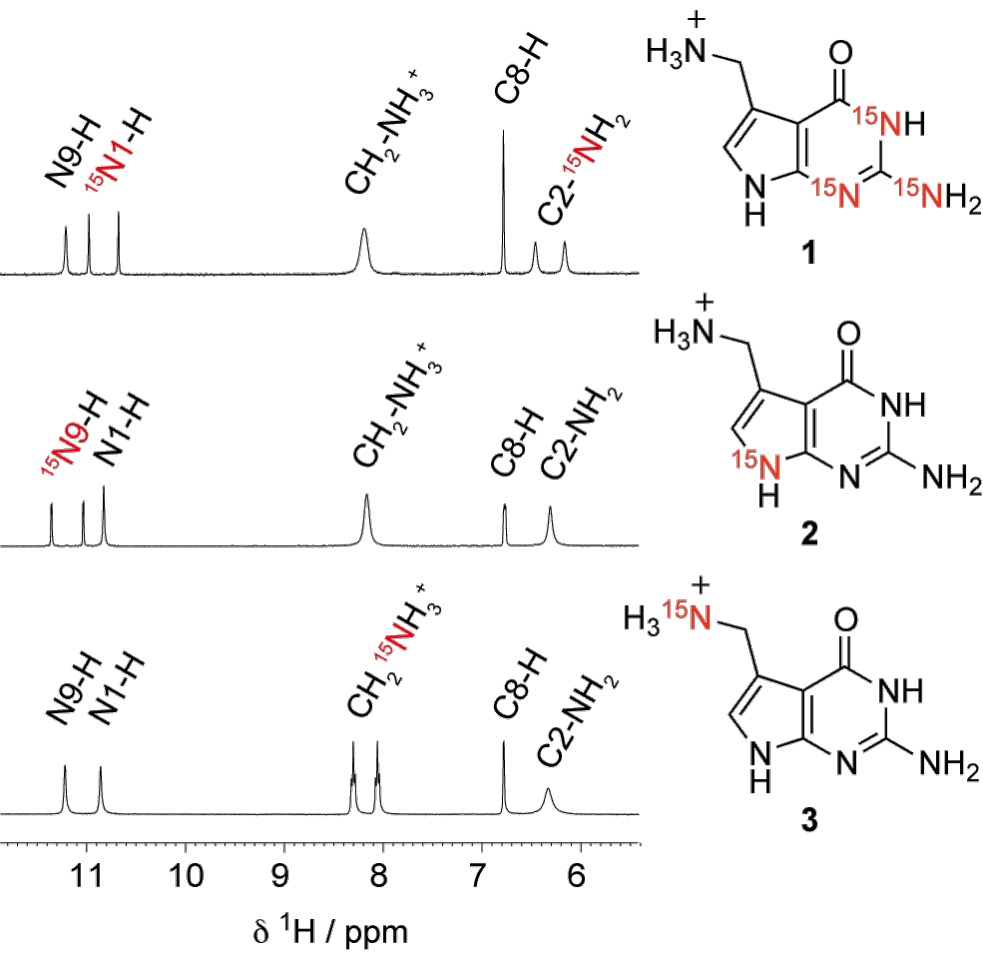
Comparison of ^1^H NMR spectra of the preQ_1_ bases **1**, **2** and **3** with complementary ^15^N labeling patterns. Conditions: *c*_preQ1 base_ = 1 mM; DMSO*-d*_6_, 298 K.

Finally, a direct comparison of ^1^H NMR spectra of the three ^15^N labeled preQ_1_ bases synthesized here is provided in Figure 1.

## Conclusion

In this short note, an efficient and cost-minimizing route for ^15^N labeled preQ_1_ base derivatives has been described. The synthesis of the pyrrolo[2,3-*d*]pyrimidine ring system is based on the cyclocondensation reaction between α-bromoaldehydes and 2,6-diaminopyrimidin-4-ones and utilizes [^15^N]-KCN, [^15^N]-phthalimide, and [^15^N_3_]-guanidine for ^15^N sources to achieve three complementary labeling patterns that cover all five nitrogen atoms of preQ_1_ base. The new derivatives carry the potential for modern NMR spectroscopic applications to study the recognition process of these small molecules with RNA aptamer domains from the three preQ_1_ riboswitch classes known to this date [4–5,18].

## Experimental

**General.**Chemical reagents and solvents were purchased from commercial suppliers (Sigma-Aldrich) and used without further purification. Organic solvents for reactions were dried overnight over freshly activated molecular sieves (4 Å). The reactions were carried out under argon atmosphere. Analytical thin-layer chromatography (TLC) was carried out on Marchery-Nagel Polygram SIL G/UV254 plates. Column chromatography was carried out on silica gel 60 (70–230 mesh). Reversed-phase column chromatography was performed on a GE Healthcare Äktaprime system using a commercial Götec-Labortechnik GmbH 310-25 LiChroprep RP-18 (40–63 µm) column (Merck Lobar compatible). The LC separation was monitored by ultraviolet (UV) detection at 280 nm. Solvent systems were as described below for the individual compounds. ^1^H and ^13^C NMR spectra were recorded on Bruker DRX 300 MHz and Bruker Avance II+ 600 MHz instruments. The chemical shifts (δ) are reported relative to tetramethylsilane (TMS) and referenced to the residual proton or carbon signal of the deuterated solvent: CDCl_3_ (7.26 ppm), DMSO-*d*_6_ (2.49 ppm), for ^1^H NMR spectra; CDCl_3_ (77.0 ppm) or DMSO-*d*_6_ (39.5 ppm) for ^13^C NMR spectra. ^1^H and ^13^C assignments are based on COSY and HSQC experiments. MS experiments were performed on a Bruker 7T FT-ICR instrument with an electrospray ion source. Samples were analyzed in the positive-ion mode.

**[^15^N1,^15^N3,H_2_^15^N(C2)]-2,6-Diaminopyrimidin-4(3*H*)-one (6).** To a solution of methyl cyanoacetate (**4**, 90 µL, 1.02 mmol) and [^15^N_3_]-guanidine hydrochloride (**5**, 100 mg, 1.02 mmol) in methanol (6.6 mL) was added dropwise NaOCH_3_ (0.53 g, 9.85 mmol) in methanol (4.1 mL). After the addition was complete, the mixture was refluxed for 10 hours and allowed to cool to room temperature. The mixture was filtrated, and the filtrate evaporated to dryness. The residue was redissolved in water (1 mL) at 90 °C. The yellow solution was then acidified to pH 6 by acetic acid. The crude product was purified by reversed-phase (C18) column chromatography (eluent A: water, eluent B: acetonitrile; 0–15% B in 40 min, 5 mL/min) as eluent to yield 46 mg of compound **6** (35%) as a yellow solid. ^1^H NMR (300 MHz, DMSO-*d*_6_) δ 4.41 (s, 1H, CH), 5.82 (s, 2H, NH_2_), 6.70 (d, *J* = 89.03 Hz, 2H, ^15^NH_2_), 7.91 (d, *J* = 89.51 Hz, 1H, ^15^NH) ppm.

**[^15^N1,^15^N3,H_2_^15^N(C2)]-2-[(2-Amino-4,7-dihydro-4-oxo-1*H*-pyrrolo[2,3-*d*]pyrimidin-5-yl)methyl]-1,3-dihydro-2*H*-isoindole-1,3-dione (8).** Compound **6** (46 mg, 0.37 mmol) and α-bromoaldehyde **7** [15] (100 mg, 0.37 mmol) were suspended in a mixture of acetonitrile and water (1.6 mL; 1:1). Sodium acetate trihydrate (97 mg, 0.72 mmol) was added, and the suspension stirred at 40 °C. After 10 minutes, all solids were disolved and a yellow solution was obtained which rapidly turned into a suspension again, indicating that product **8** started to precipitate. The mixture was stirred for 4 hours, then cooled to room temperature and filtered. The residue was dried under reduced pressure to yield 70 mg of compound **8** (63%) as yellow solid. ^1^H NMR (300 MHz, DMSO-*d*_6_) δ 4.84 (s, 2H, CH_2_-N), 6.16 (d, *J* = 88.96 Hz, 2H, ^15^NH_2_-C2), 6.36 (s, 1H, H-C8), 7.85–7.87 (m, 4H, arom H), 10.42 (d, *J* = 89.21 Hz, 1H, ^15^N1-H), 10.81 (s, 1H, NH) ppm.

**Trifluoroacetate salt of [^15^N1,^15^N3,H_2_^15^N(C2)]-7-(aminomethyl)-7-deazaguanine (1).** Compound **8** (70 mg, 0.22 mmol) was added to a solution containing hydrazine hydrate (111 µL, 2.2 mmol) and ethanol (3 mL). The mixture was refluxed overnight and evaporated to dryness. The residue was purified by reversed-phase (C18) column chromatograpy (eluent A: 1% trifluoroacetic acid in H_2_O; eluent B: acetonitrile; 0–20% B in 50 min, 4 mL/min) to yield 49 mg of compound **1** (75%; calculated as mono TFA salt) as a light yellow solid. ^1^H NMR (300 MHz, DMSO-*d*_6_) δ 4.01 (s, 2H,CH_2_), 6.30 (d, *J* = 89.1 Hz, 2H, ^15^NH_2_-C2), 6.78 (s, 1H, H-C8), 8.18 (s, 3H, NH_3_^+^), 10.82 (d, *J* = 89.6 Hz, 1H, ^15^N1-H), 11.20 (s, 1H, NH) ppm; ^13^C NMR (75 MHz, D_2_O) δ 34.57 (CH_2_NH_3_^+^), 98.32 (C7), 110.29 (C5), 113.88 (C2), 118.55 (C8), 141.05 (C4), 150.60 (C6=O), 159.09 (COO^−^), 162.18 (CF_3_) ppm; HRMS–ESI *m*/*z*: [M + H]^+^ calcd for C_7_H_9_N_2_^15^N_3_O, 183.07909; found, 183.07865.

**UV spectroscopic analysis of unlabeled 7-(aminomethyl)-7-deazaguanine (preQ_1_ base):** UV (H_2_O) λ_max_ (ε) = 218 (13630), 258 (7940) nm. For comparison, see UV spectroscopic data and extinction coefficients of 7-deazaguanine, *N*9-methyl-7-deazaguanine and preQ_1_ nucleoside in references [19–21].

## Supporting Information

^1^H and ^13^C NMR spectra are provided in Supporting Information File 1. Synthetic procedures for the syntheses of compounds **2**, **3**, **10**, **11**, **13**, **14**, **17–19**, and **21**.

File 1 Synthetic procedures and NMR spectra of the most typical compounds.
