# Supplementary material for: Syntheses of 15N-labeled pre-queuosine nucleobase derivatives
Source: Beilstein J Org Chem. 2014 Aug 18;10:1914–8. doi: 10.3762/bjoc.10.199 (PMC4168690; doi:10.3762/bjoc.10.199)
Supplement: File 1 — Synthetic procedures and NMR spectra of the most typical compounds. [file Beilstein_J_Org_Chem-10-1914-s001.pdf]

Supporting Information

for

Syntheses of ^{15}N-labeled pre-queuosine nucleobase derivatives

Jasmin Levic[1] and Ronald Micura*[1]

Address: [1]Institute of Organic Chemistry, University of Innsbruck and Center for Molecular Biosciences Innsbruck, Innrain 80–82, 6020 Innsbruck, Austria

Email: Ronald Micura* - ronald.micura@uibk.ac.at

* Corresponding author

Synthetic procedures and NMR spectra of the most typical compounds

Experimental procedures

[^{15}N]-2-Cyanoacetic acid (**10**). 2-Bromoacetic acid **9** (214 mg, 1.543 mmol) was dissolved in water (0.6 mL). To this solution, Na_2CO_3 (104 mg, 1.32 mmol) dissolved in water was added, until the pH value was 9. Then, [^{15}N]-KCN (100 mg, 1.513 mmol) was added and the mixture stirred at 80 °C for 3 hours, then at room temperature for 20 hours. Concentrated hydrochlorid acid was added until pH 1. The solvents were evaporated, and the residue was dried under reduced pressure for 30 minutes, followed by the extraction of the residue with diethyl ether (50 mL). The solvents were evaporated, and the resulting yellow oil was dried under reduced pressure, to yield 117 mg of compound **10** (90%) as yellow crystalline powder. ^{1}H NMR (300 MHz, DMSO-d_6): δ 3.86 (d, J=1.38 Hz, 2H, CH_2) ppm. ^{13}C NMR (75 MHz, DMSO-d_6): δ 24.66 (d, J=2.85 Hz, CH_2); 115.69 (d, J=17.56 Hz, C^{15}N); 165.74 (CO) ppm.

Ethyl [^{15}N]-2-cyanoacetate (**11**). [^{15}N]-2-Cyanoacetic acid **10** (117 mg, 1.358 mmol) was dissolved in dichloromethane (30 mL). Ethanol (400 µL, 6.791 mmol) and 3 drops of concentrated H_2SO_4 were added, and the mixture was refluxed for 18 hours. The mixture was extracted with an aqueous solution of 10% (w/w) $NaHCO_3$. The aqueous layer was then extracted twice with dichloromethane (50 mL). The organic layer was dried over Na_2SO_4, filtrated and evaporated. The residue was dried under reduced pressure to yield 142 mg of compound **11** (92%) as a light yellow oil. ^{1}H NMR (300 MHz, $CDCl_3$): δ 1.32 (t, J=7.15 Hz, 3H, CH_3); 3.45 (d, J=1.49 Hz, 2H, CH_2); 4.28 (q, J=7.18, 2H, O-CH_2) ppm. ^{13}C NMR (75 MHz, $CDCl_3$): δ 13.55 (CH_3), 24.31 (d, J=3.27 Hz, CH_2), 62.60 (O-CH_2); 112.46 (d, J=17.63 Hz, C^{15}N) ppm.

[$H_2{}^{15}N(C6)$]-2,6-Diaminopyrimidin-4(3H)-one (**13**). To a solution of compound **11** (142 mg, 1.247 mmol) and guanidine hydrochloride **12** (119 mg, 1.247 mmol.) in MeOH (8 mL), a solution of NaOMe (0.65 g, 12.1 mmol) in MeOH (5 mL) was added and the

mixture refluxed for 10 hours. The solvents were evaporated, and the residue dissolved in boiling water. Then, acetic acid was added until pH 6. This solution was then purified by reversed-phase (C18) column chromatography (eluant A: H_2O; eluant B: acetonitrile; 0–15% B in 40 min, 5 mL/min) to yield 62 mg of compound **13** (40%) as a yellow solid. ^{1}H NMR (75 MHz, DMSO-d_6): δ 4.44 (s, 1H, CH); 5.87 (d, J=88.29 Hz, 2H, $^{15}NH_2$); 6.14 (s, 2H, NH_2) ppm.

[^{15}N7]-2-[(2-Amino-4,7-dihydro-4-oxo-1H-pyrrolo[2,3-d]pyrimidin-5-yl)methyl]-1,3-dihydro-2H-isoindole-1,3-dione (**14**). Analogous procedure as for compound **8**. Yield: 78 mg (52%) of compound **14** as a slightly yellow solid. This product was directly used for the next step without further characterization.

Trifluoroacetate salt of [^{15}N9]-7-(aminomethyl)-7-deazaguanine (**2**). Analogous procedure as for compound **1**. Yield: 68 mg of compound **2** (92%; calculated as mono TFA salt) as a slightly yellow solid. ^{1}H NMR (300 MHz, DMSO-d_6): δ 4.01-4.02 (m, 2H, CH_2); 6.31 (s, 2H, NH_2-C2); 6.76-6.78 (m, 1H, C8-H); 8.17 (s, 3H, NH_3^+); 10.83 (s, 1H, N1-H); 11.20 (dd, J_1=2.21, J_2=94.44, 1H, ^{15}N9-H) ppm. ^{13}C NMR (75 MHz, D_2O: δ 34.54 ($CH_2NH_3^+$); 98.40 (d, J=5.00 Hz, C7); 110.42 (C5); 114.00 (C2); 118.68 (d, J=12.70 Hz, C8-^{15}N); 140.25 (d, J=20.37,C4); 150.37 (C6=O); 159.00 (COO^-); 162.16 (CF_3). ESI-HRMS: m/z of $[M+H]^+$ of $C_7H_9N_4{}^{15}NO$, calcd 181.08502; found 181.08519.

[^{15}N]-2-(3-Hydroxypropyl)-1,3-dioxo-1,3-dihydro-2H-isoindole (**17**). 3-Chloropropanol **15** (0.57 mL, 6.75 mmol), [^{15}N]-phthalimide **16** (1 g, 6.75 mmol) and K_2CO_3 (1.4 g, 10.13 mmol) in DMF (5 mL) were stirred at 70 °C overnight and allowed to cool to room temperature. Thereafter, water (20 mL) was added and extracted with ethylacetate (three times with 30 mL). The organic layer was washed with water and with a saturated solution of NaCl, dried over Na_2SO_4, filtered and evaporated. The residue was then purified by column chromatography on SiO_2 (ethylacetate/hexane

1:1) to yield 0.66 g of compound **17** (47%) as a white solid. ^{1}H NMR (300 MHz, $CDCl_3$): δ 1.83-1.88 (m, 2H, CH_2); 3.60 (t, *J*=6.32 Hz, 2H, CH_2-OH); 3.81 (t, *J*=5.89 Hz, 2H, CH_2-^{15}N); 7.68-7.71 (m, 2H, arom); 7.80-7.81 (m, 2H, arom) ppm. ^{13}C NMR (75 MHz, $CDCl_3$): δ 30.91 (C2´); 33.96 (d, *J*=9.33 Hz, C1´); 58.70 (C3´); 122.90 ($C(3,6)_{arom}$); 131.52 ($C(2,7)_{arom}$); 133.65 ($C(4,5)_{arom}$); 168.33, 168.51 (2 CO) ppm.

[^{15}N]-3-(1,3-Dioxo-1,3-dihydro-2H-isoindol-2-yl)propanal (**18**). [^{15}N]-3-Phthalimido-propan-1-ol **17** (0.4 g, 1.94 mmol) was dissolved in dichloromethane (13 mL). To this solution Dess-Martin Periodinane (1.23 g, 2.91 mmol) was added and the mixture was stirred at room temperature. The progress of the reaction was analyzed by thin layer chromatography (ethylacetate/hexane 1:1). The reaction was completed after 2 to 3 hours. Then, dichloromethane (26 mL) was added and the solution washed consecutively with saturated aqueous solutions of $Na_2S_2O_3$, $NaHCO_3$ and finally sodium chloride. The organic layer was then dried over Na_2SO_4, filtered and evaporated to dryness. The residue was purified on column chromatography on SiO_2 (ethylacetate/hexane 1/1) to yield 0.33 g of **17** (85%) as a white solid. ^{1}H NMR (300 MHz, $CDCl_3$): δ 2.85-2.90 (m, 2H, CH_2); 4.04 (t, *J*=7.00 Hz, 2H, CH_2-^{15}N); 7.74-7.84 (m, 4H, arom); 9.82 (s, 1H, CHO) ppm. ^{13}C NMR (75 MHz, $CDCl_3$): δ 31.18 (d, *J*= 10.33 Hz, ^{15}N-CH_2); 41.93 (CH_2); 123.00 ($C(3,6)_{arom}$); 131.50 und 131.60 ($C(2,7)_{arom}$); 133.70 ($C(4,5)_{arom}$); 167.50-167.66 (2 CO); 199.00 (CHO) ppm.

[^{15}N]-2-Bromo-3-(1,3-dioxo-1,3-dihydro-2H-isoindol-2-yl)propanal (**19**). To a solution of compound **18** (0.3 g, 1.48 mmol) in acetonitrile (2.5 mL) was added 5,5-dibromobarbituric acid [1] (0.25 g, 0.89 mmol), and the mixture was refluxed for 2 hours. After completion of the reaction, water was added thereto, followed by extraction with ethyl acetate. The organic layer was dried over Na_2SO_4 , filtered and evaporated. The residue was then adsorbed on SiO_2 and purified by column chromatography (ethylacetate/hexane 1/1) to yield 0.19 g of compound **19** (45%) as

a white solid. ^{1}H NMR (300 MHz, $CDCl_3$): δ 4.2 (d, J=7.06 Hz, 2H, CH_2-^{15}N); 4.67 (t, J= 7.27 Hz, CHBr); 7.73-7.87 (m, 4H, arom H); 9.52 (d, J= 2.64 Hz, 1H, CHO) ppm. ^{13}C NMR (75 MHz, $CDCl_3$): δ 38.61 (d, J= 10.33 Hz, ^{15}N-CH_2); 49.93 (CHBr); 124.15 ($C(3,6)_{arom}$); 131.81 ($C(2,7)_{arom}$); 134.80 ($C(4,5)_{arom}$); 167.76 (2 CO); 191.39 (CHO) ppm.

[H_2^{15}N-C5']-2-[(2-Amino-4,7-dihydro-4-oxo-1H-pyrrolo[2,3-d]pyrimidin-5-yl)methyl]-1,3-dihydro-2H-isoindole-1,3-dione (**21**). Analogous procedure as for compound **8**. Yield: 90 mg (58%) of compound **21** as a slightly yellow solid. This product was directly used for the next step without further characterization.

Trifluoroacetate salt of [H_2^{15}N(C7')]-7-(aminomethyl)-7-deazaguanine (**3**). Analogous procedure as for compound **1**. Yield: 35 mg of compound **3** (82%; calculated as mono TFA salt) as a slightly yellow solid. ^{1}H NMR (300 MHz, DMSO-d_6): δ 4.01-4.03 (m, 2H,CH_2); 6.33 (s, 2H, NH_2-CH_2); 6.77 (s, 1H, H-C(8)); 8.17 (dt, J_1= 5.72 Hz, J_2= 73.32 Hz, 3H, $^{15}NH_3^+$); 10.84 (s, 1H, N1-H); 11.20 (s, 1H, N9-H) ppm. ^{13}C NMR (75 MHz, D_2O): δ 35.03 (d, J=5.47 Hz, $CH_2{}^{15}NH_3^+I$); 99.02 (C7); 111.02 (C5); 114.42 (C2); 119.22 (C8); 140.30 (C4); 150.84 (C6=O); 159.57 (COO^-); 162.71 (CF_3). ESI-HRMS: m/z of $[M+H]^+$ of $C_7H_9N_4{}^{15}NO$, calcd 181.08502; found 181.08495.

NMR spectra

*[^{15}N1,^{15}N3,^{15}N(C2)]-2,6-Diaminopyrimidin-4(3*H*)-one* (**6**)

^{1}H NMR (300 MHz, DMSO-d_6)

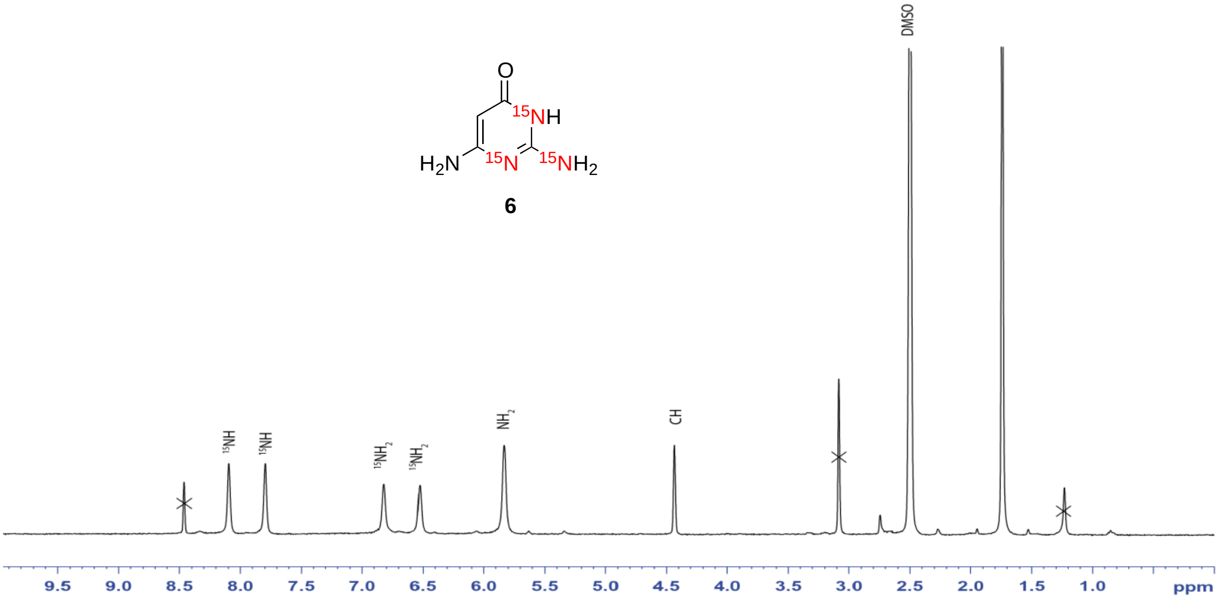

Trifluoroacetate salt of [^{15}N1,^{15}N3,^{15}N(C2)]-7-(aminomethyl)-7-deazaguanine (**1**)

^{1}H NMR (300 MHz, DMSO-d_6)

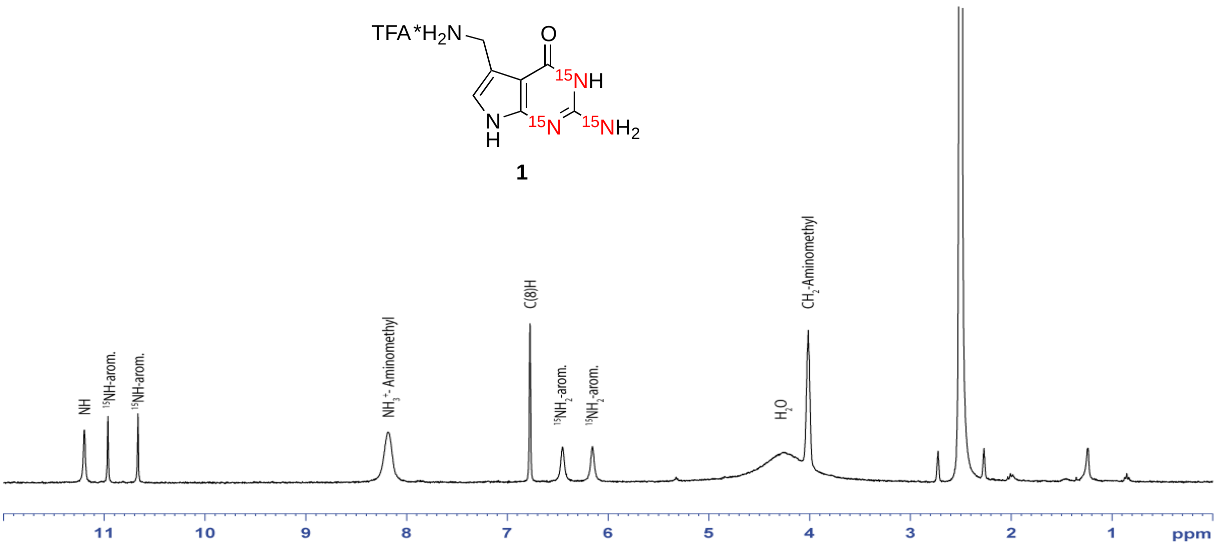

Trifluoroacetate salt of [15N1,15N3,15N(C2)]-7-(aminomethyl)-7-deazaguanine (**1**)

^{13}C NMR (75 MHz, D_2O)

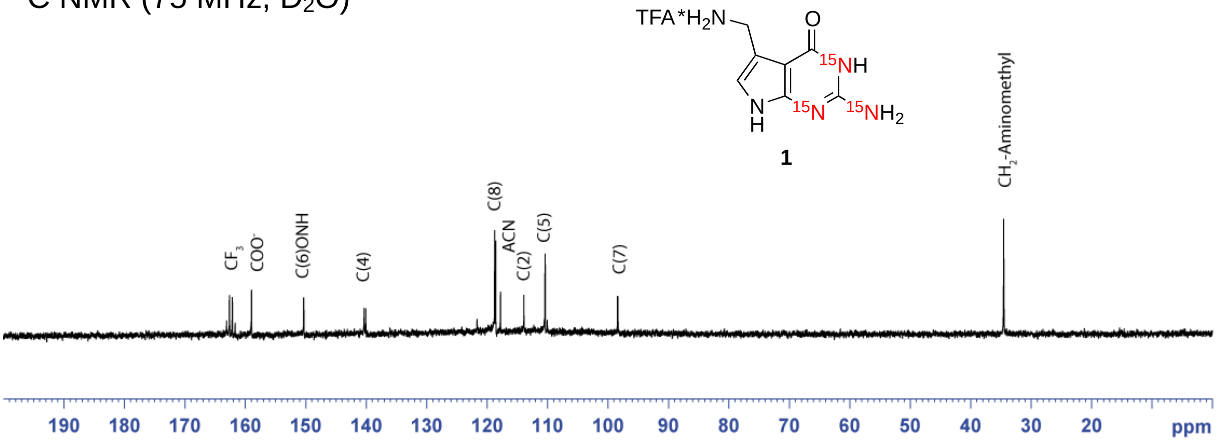

[15N]-2-Cyanoacetic acid (**10**)

^{1}H NMR (300 MHz, DMSO-d_6)

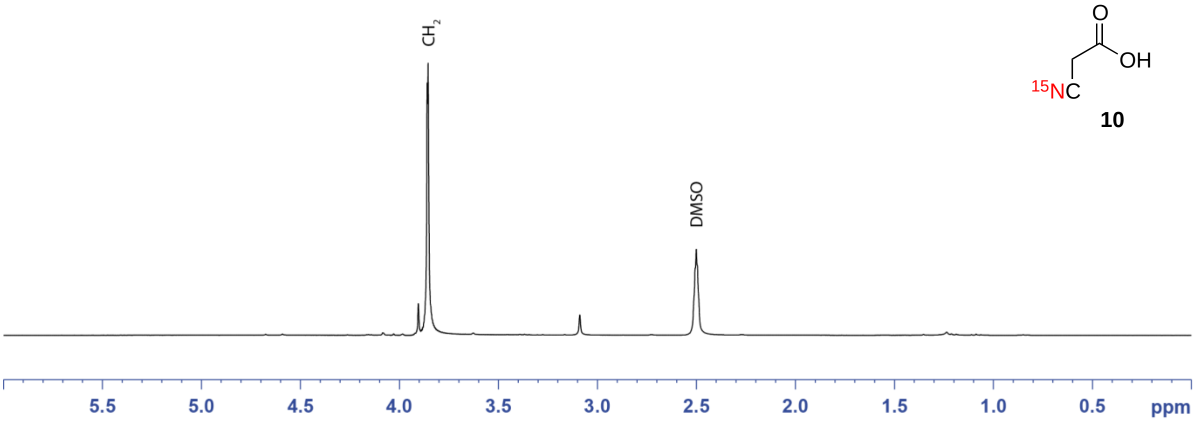

^{13}C NMR (75 MHz, DMSO-d_6)

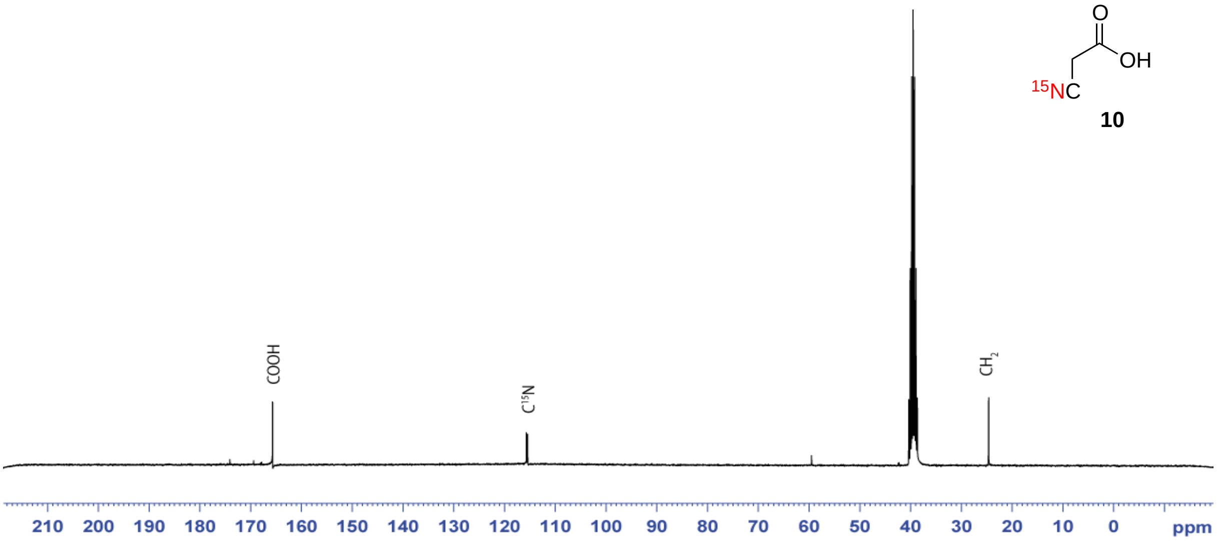

Ethyl [^{15}N]-2-cyanoacetate (**11**)

NMR (300 MHz, $CDCl_3$)

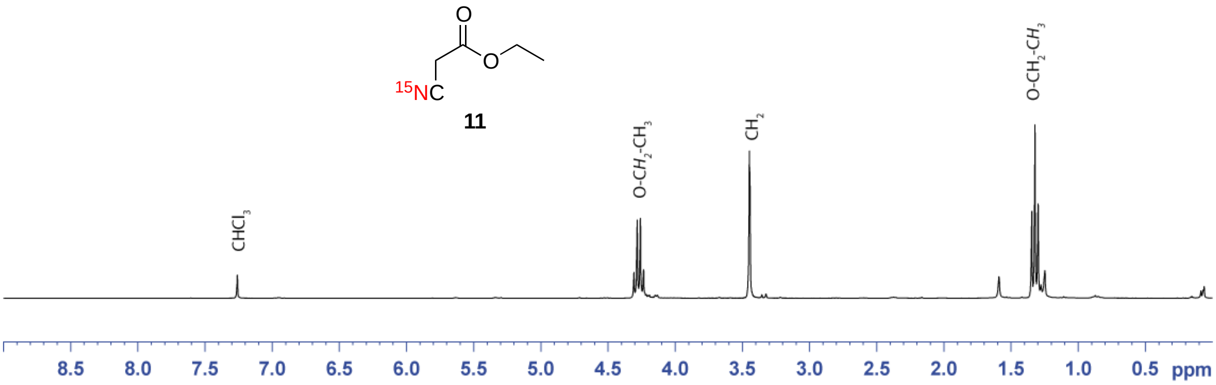

^{13}C NMR (75 MHz, $CDCl_3$)

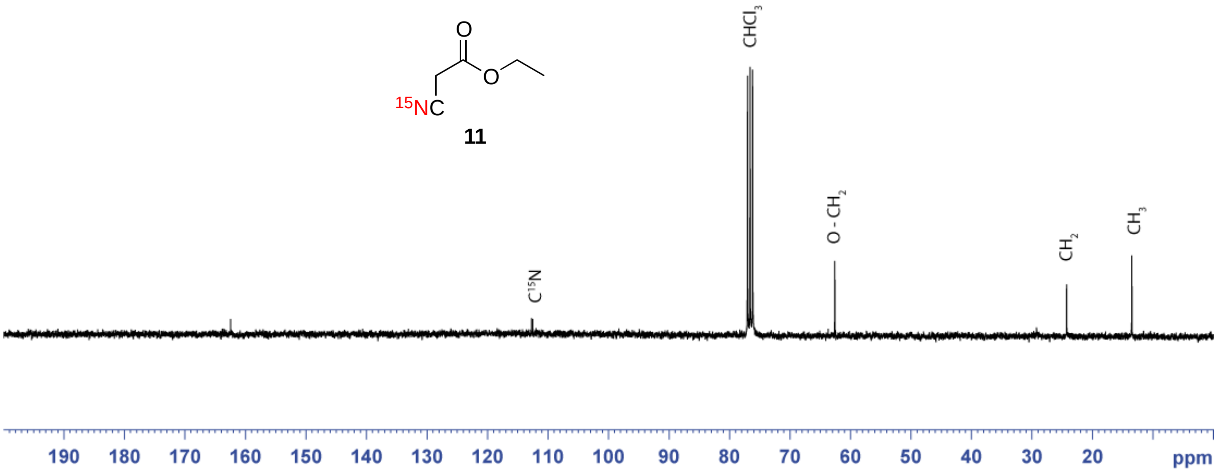

[^{15}N(C6)]-2,6-Diaminopyrimidin-4(3H)-one (**13**)

^{1}H NMR (300 MHz, DMSO-d_6)

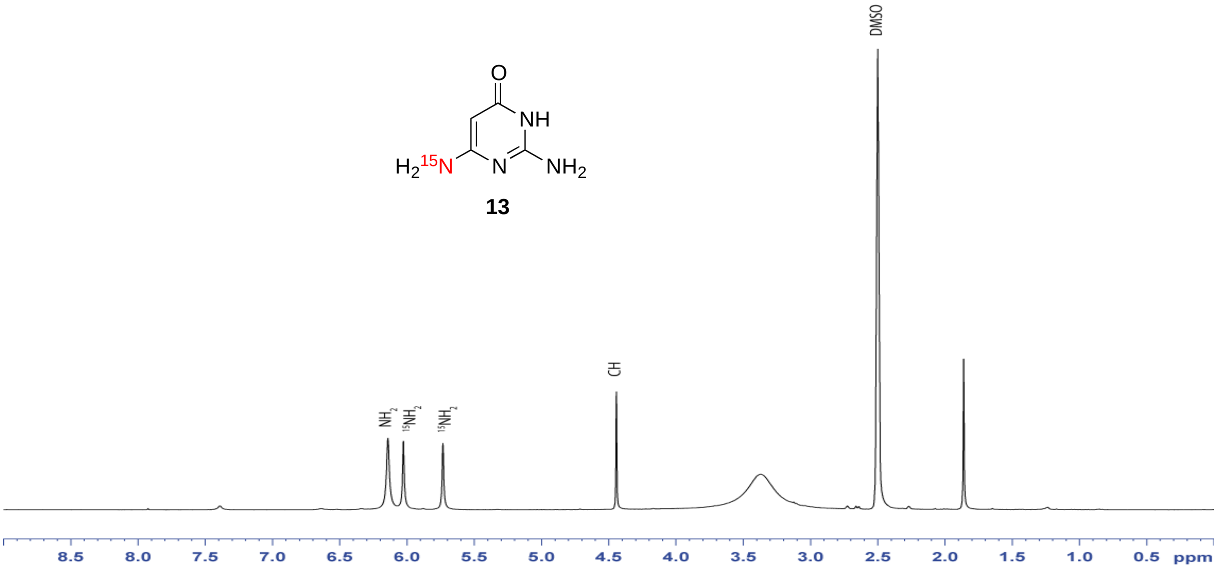

Trifluoroacetate salt of [^{15}N9]-7-(aminomethyl)-7-deazaguanine (**2**)

^{1}H NMR (300 MHz, DMSO-d_6)

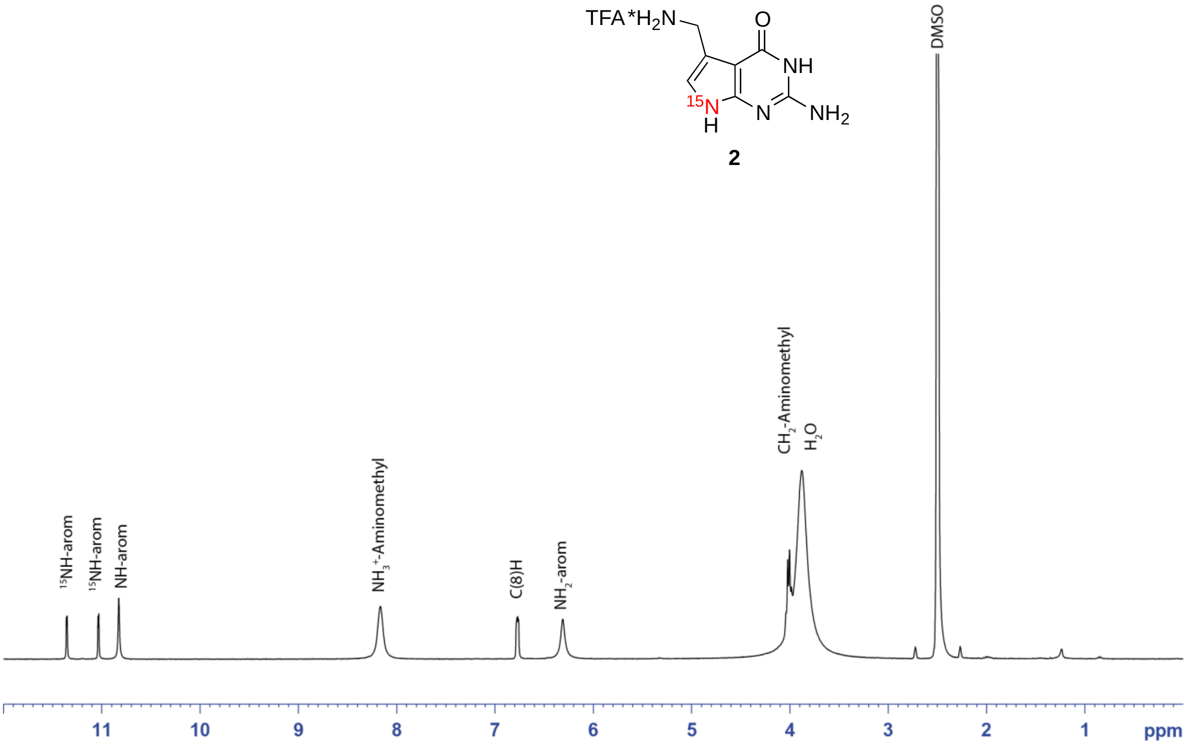

^{13}C NMR (75 MHz, D_2O)

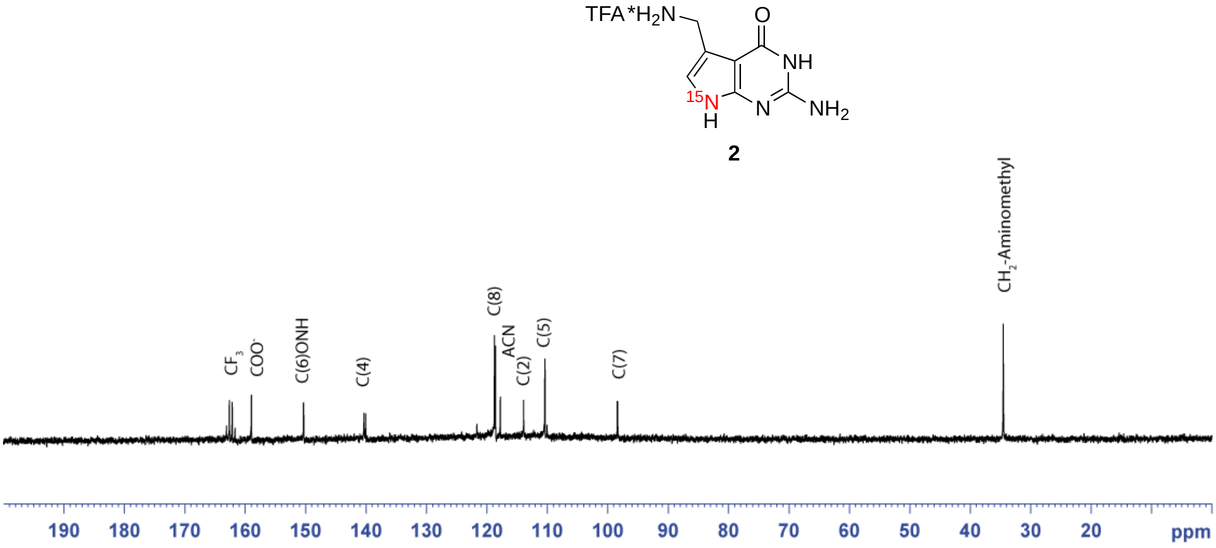

[^{15}N]-2-(3-Hydroxypropyl)-1,3-dioxo-1,3-dihydro-2H-isoindole (**17**)

^{1}H NMR (300 MHz, $CDCl_3$)

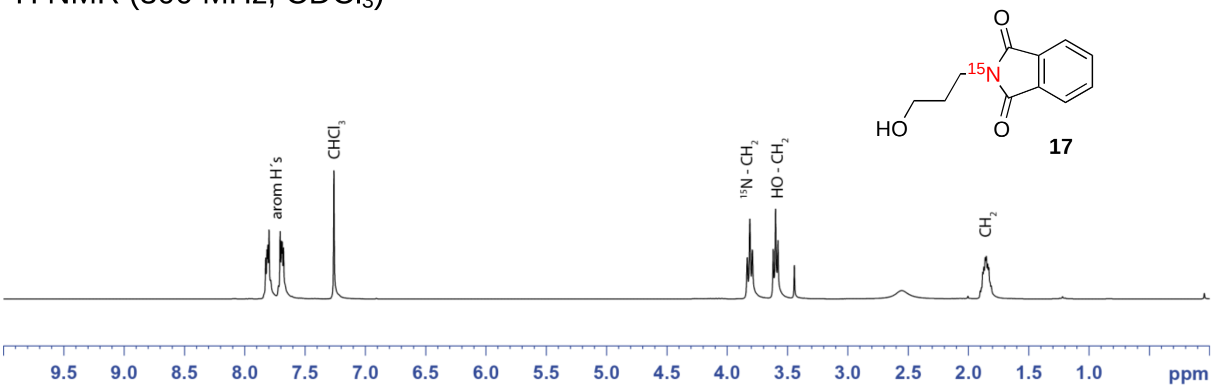

^{13}C NMR (75 MHz, $CDCl_3$)

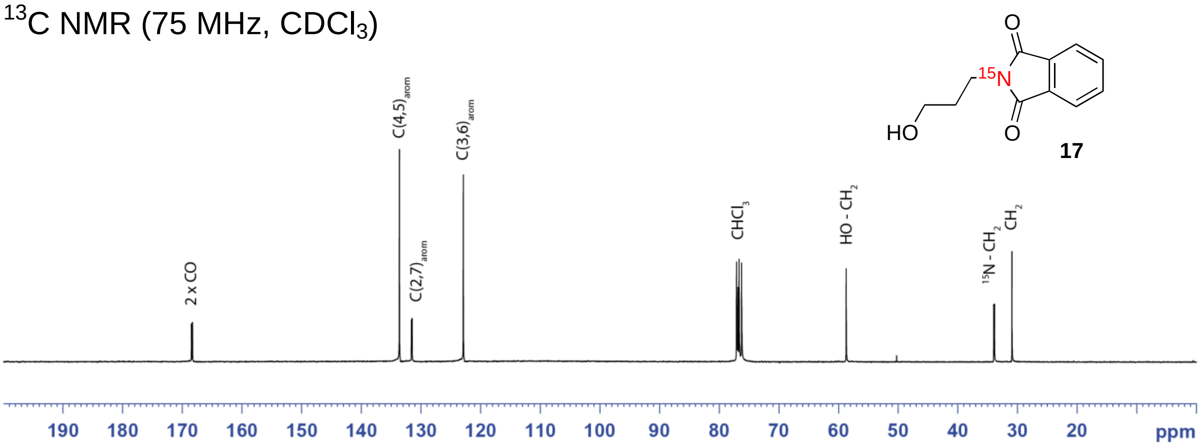

[^{15}N]-3-(1,3-Dioxo-1,3-dihydro-2H-isoindol-2-yl)propanal (**18**)

^{1}H NMR (300 MHz, $CDCl_3$)

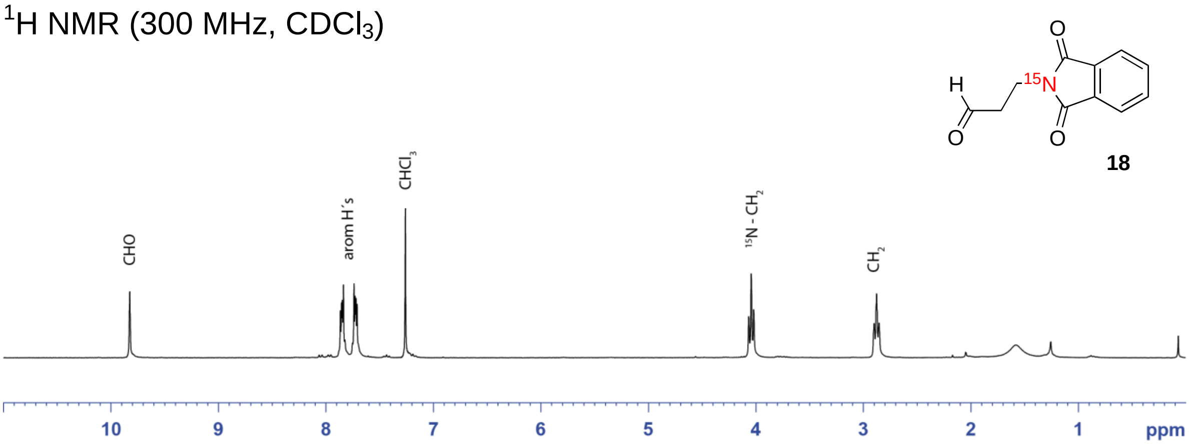

[15N]-3-(1,3-Dioxo-1,3-dihydro-2H-isoindol-2-yl)propanal (**18**)

^{13}C NMR (75 MHz, $CDCl_3$)

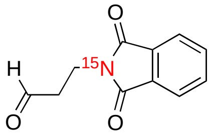

18

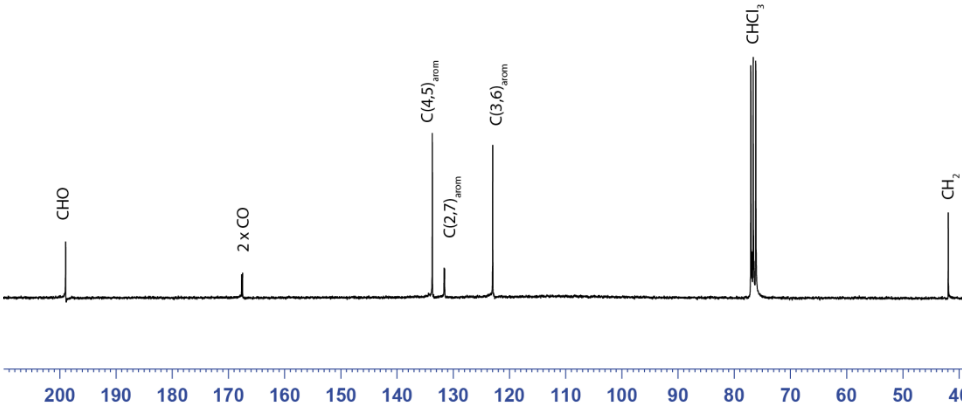

[15N]-2-Bromo-3-(1,3-dioxo-1,3-dihydro-2H-isoindol-2-yl)propanal (**19**)

^{1}H NMR (300 MHz, $CDCl_3$)

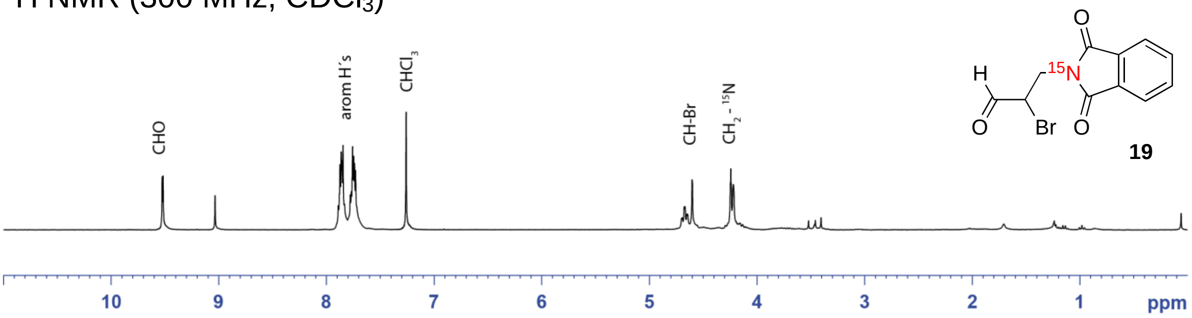

19

^{13}C NMR (75 MHz, $CDCl_3$)

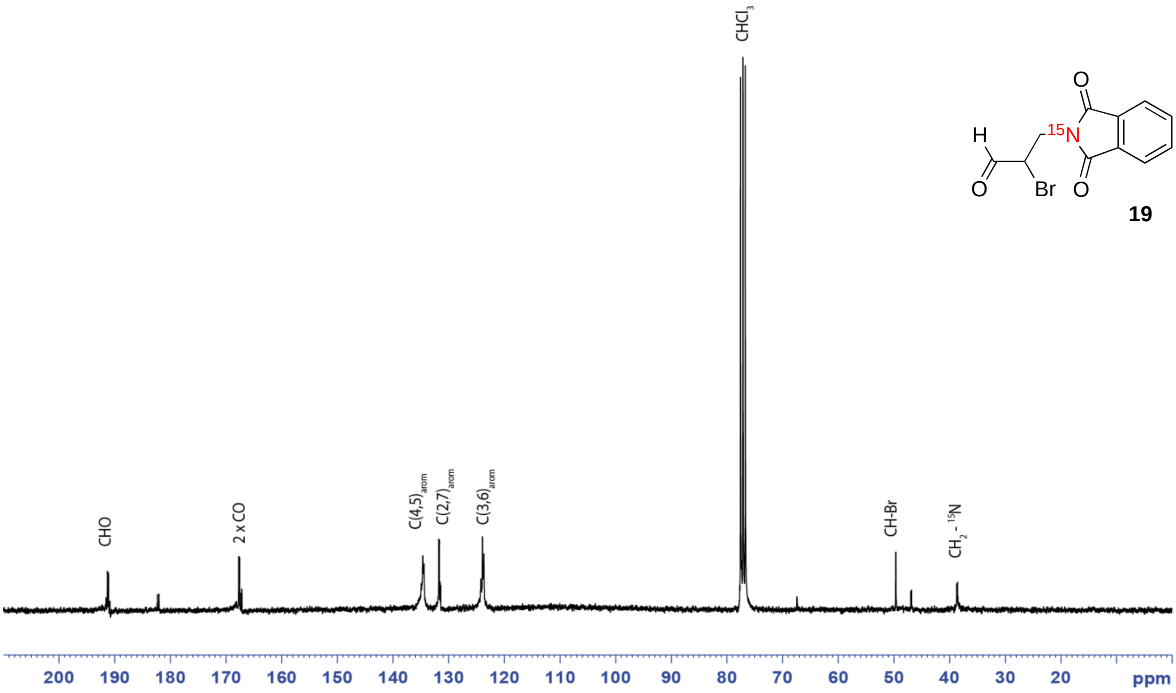

19

Trifluoroacetate salt of [^{15}N(C7')]-7-(aminomethyl)-7-deazaguanine (**3**)

^{1}H NMR (300 MHz, DMSO-d_6)

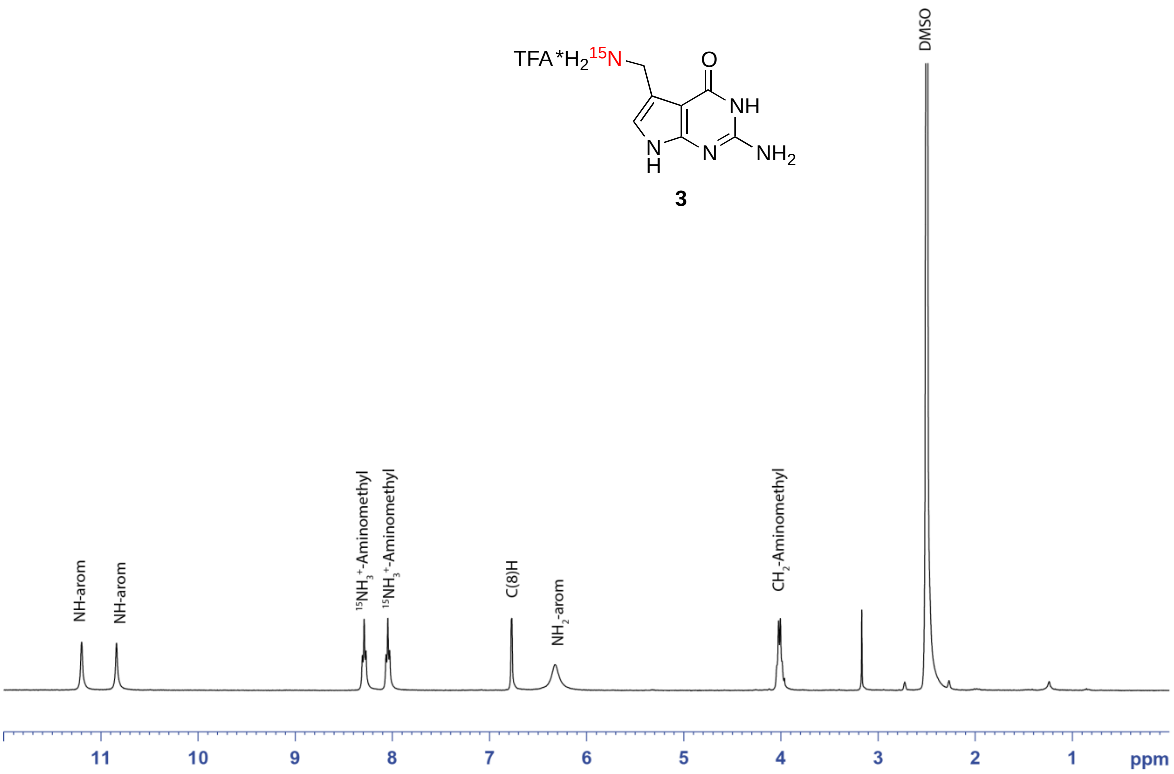

^{13}C NMR (75 MHz, D$_2$O)

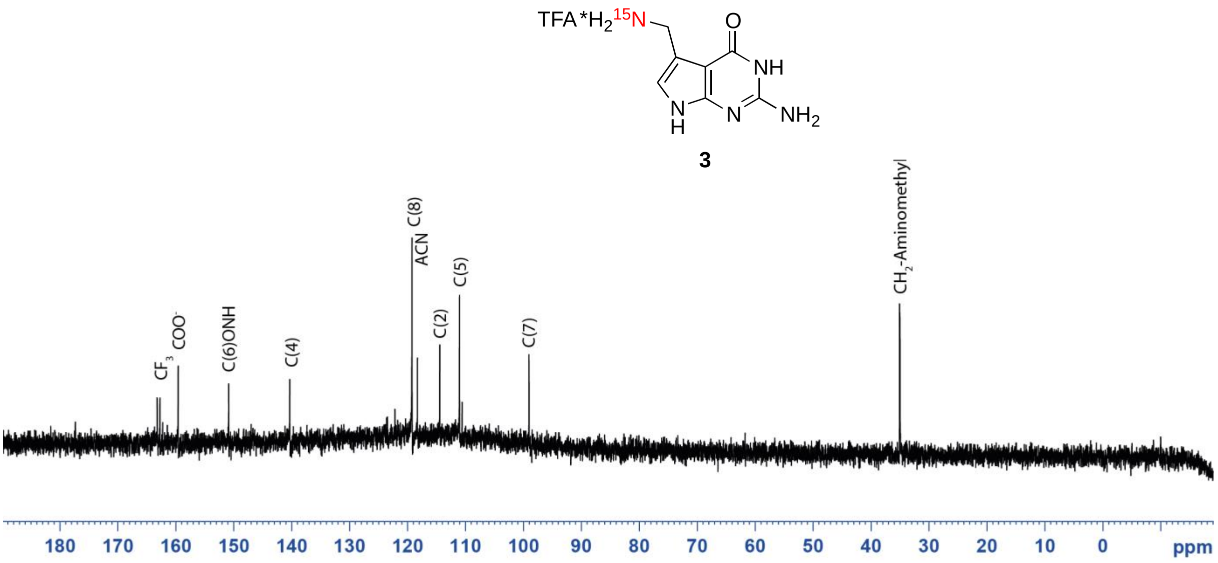

UV spectra of preQ$_1$ (TFA salt)

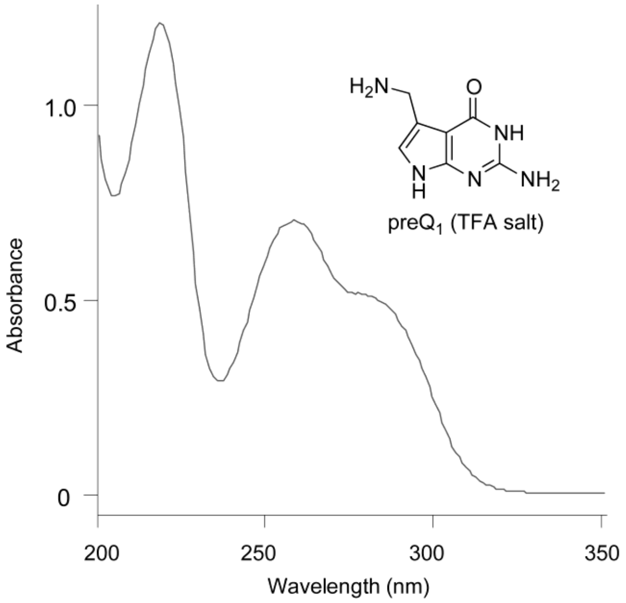

UV spectra of preQ$_1$ (TFA salt) in H_2O; λ_{max} (ε) = 218 (13630), 258 (7940).

Reference

1. Yamaguchi, H.; Thiazolimine compound and oxazolimine compound; Sumitomo Pharmaceuticals Co. Ltd. Osaka, Eur. Pat. Appl. EP1640369 A1, Mar 29, 2006, pp. 26–27.
